# Multi-Omics Analysis Reveals Differences in Pork Quality and Skeletal Muscle Characteristics Between Tianfu Black and DLY Pigs

**DOI:** 10.3390/foods15213932

**Published:** 2026-11-02

**Authors:** Zongze He, Bing Yu, Quyuan Wang, Huifen Wang, Hanna Dai, Daiwen Chen

**Affiliations:** 1 Institute of Animal Nutrition, Sichuan Agricultural University, Chengdu 611130, China; xzongze@outlook.com (Z.H.); hanndai@163.com (H.D.); 2 Key Laboratory for Animal Disease-Resistance Nutrition of China Ministry of Education, Sichuan Agricultural University, Chengdu 611130, China

**Keywords:** Tianfu Black pig, meat quality, intramuscular fat, muscle fiber type, multi-omics, water-holding capacity

## Abstract

This study compared pork quality and muscle molecular profiles between Tianfu Black (TFB) and Duroc × (Landrace × Yorkshire) (DLY) pigs under a common-age finishing design. Forty-eight barrows (24 per group) were studied from 153 to 195 days of age; eight pigs per group contributed terminal measurements and six contributed each omics dataset. TFB pigs had lower drip loss, cooking loss, and shear force and higher intramuscular fat content, alongside higher oxidative MyHC markers. TFB also had lower serum triglyceride and total cholesterol concentrations despite greater fat deposition. DLY muscle had higher p-mTOR/mTOR and p-4EBP1/4EBP1 ratios. Transcriptomic analysis identified 989 differentially expressed genes, with 673 higher in TFB and 316 higher in DLY. Pathway-level analysis showed TFB enrichment of oxidative phosphorylation and protein export, and DLY enrichment of carbohydrate- and cholesterol-related pathways, cytoskeletal organization, and motor proteins. Metabolomic analysis identified 16 features that differed after multiple-testing correction, including putatively annotated 1-methylhistidine and N-acetyl-1-methylhistidine, both higher in TFB. Histidine metabolism was enriched in TFB after multiple-testing correction. *CARNMT1* expression was also higher in TFB, placing a transcriptomic difference within the carnosine–anserine branch of histidine-related metabolism. Together, the muscle fiber, mitochondrial, and histidine-related profiles provide a molecular context for the observed pork quality differences.

## 1. Introduction

Improving pork quality traits while maintaining carcass yield and leanness is an important objective in pig production. Duroc × (Landrace × Yorkshire) (DLY) pigs are widely used commercial crossbreds selected for lean yield [1]. Comparisons with indigenous and black pig production types have reported differences in intramuscular fat and water-holding characteristics [2,3,4,5]. Tianfu Black (TFB) is a locally developed black pig production type of interest for quality-oriented production [6]. Comparisons of TFB and DLY that jointly characterize carcass traits, meat quality traits, muscle fiber characteristics, and molecular profiles under a common finishing period remain limited [6,7]. Such comparisons provide a basis for identifying the muscle characteristics associated with their contrasting production phenotypes.

Pork quality traits reflect muscle composition and biochemical changes during conversion of muscle to meat. Muscle fiber characteristics are associated with mitochondrial content and substrate utilization, and previous studies have related oxidative fiber characteristics to water-holding traits [8,9,10,11]. Energy metabolism, redox processes, and metabolite abundance provide additional dimensions for investigating these relationships [12,13]. An unresolved question is whether the water loss, intramuscular fat, and shear force differences between TFB and DLY are accompanied by corresponding differences in fiber markers and energy-metabolism-related molecular profiles.

Transcriptomic and metabolomic studies have identified pathways associated with muscle development and pork quality variation [14,15,16,17,18]. Profiling both molecular layers in the same production groups connects gene-expression patterns with metabolite abundance. We combined pathway-level comparison with an exploratory feature–trait screen to identify molecular candidates associated with the contrasting quality phenotypes.

Accordingly, this study compared carcass traits and meat quality between TFB and DLY pigs at a common slaughter age. We hypothesized that their contrasting meat quality profiles would be accompanied by differences in muscle fiber characteristics and energy metabolism. To examine this hypothesis, phenotypic measurements and muscle fiber and protein analyses were combined with transcriptomic and metabolomic profiling of the longissimus muscle. This approach aimed to characterize the muscle properties associated with the observed quality differences and identify molecular features and pathways for further investigation.

## 2. Materials and Methods

### 2.1. Ethics Statement

This experimental protocol was approved by the Institutional Animal Care and Use Committee of Sichuan Agricultural University in July 2024 (approval number 202411735). All procedures were conducted in accordance with institutional guidelines for the care and use of experimental animals.

### 2.2. Experimental Design

The study included 48 barrows, comprising 24 DLY pigs obtained from Sichuan Tieqilishi Animal Husbandry Science and Technology Co., Ltd. (Mianyang, China), and 24 TFB pigs obtained from Sichuan Jialin Black Pig Agricultural Science and Technology Co., Ltd. (Qionglai, Chengdu, China). Each group occupied eight pens containing three pigs each. Pens had partially slatted floors and an area of 3.6 m^2^; room temperature was maintained at 18–22 °C and relative humidity at 60–70%, under a 12 h light/dark cycle. Both groups received the basal diet described in Supplementary Table S1, with feeding at 08:00, 14:00, and 20:00 and continuous access to water. The pigs had been maintained in ongoing farm trials before entry into the present finishing-stage study at 153 days of age. Initial individual body weights at that age were 98.22 ± 4.55 kg for DLY and 72.76 ± 1.21 kg for TFB (mean ± SD; 24 pigs per group). Individual birth records and farm performance records were used to verify chronological age, confirming the present trial period from 153 to 195 days. One pig whose body weight was closest to its pen mean was selected from each pen for terminal measurements, yielding eight pigs per group. Selected pigs were numbered by pen, and animals 1–6 per group were designated for paired transcriptomic and metabolomic analyses owing to resource constraints. The omics subset was designated before meat quality assessment. Feed allowance was adjusted to maintain adequate availability, and feed offered and remaining were weighed daily per pen. Over 42 days, ADG and ADFI were calculated as pen weight gain and feed intake divided by pig-days, respectively; F/G was feed intake divided by weight gain.

### 2.3. Chemical Analysis of Feed

Dietary moisture, crude protein, crude fat, calcium, and total phosphorus were determined using AOAC methods 934.01 (oven drying at 105 °C to constant weight), 976.05 (Kjeldahl), 922.06 (Soxhlet extraction), and 985.01 A, B, and C (inductively coupled plasma spectroscopy), respectively [19]. Crude ash was determined according to GB/T 6438–2007 [20], and gross energy was measured using a Model 6400 oxygen bomb calorimeter (Parr Instrument Company, Moline, IL, USA).

### 2.4. Carcass Measurements

Pigs were electrically stunned before exsanguination to minimize pain and distress at slaughter. After removal of the head, hooves, tail, hide, and viscera (with kidneys retained), the hot carcass weight was recorded. Dressing percentage was calculated as the ratio of hot carcass weight to pre-slaughter live weight. Subsequently, each carcass was split longitudinally along the midline, and carcass length was measured. Average backfat thickness was calculated as the mean of measurements taken at the first rib, last rib, and last lumbar vertebra. Fat and muscle depths were measured at the interface of the 3rd–4th ribs, perpendicular to the skin surface, using a digital caliper. The loin muscle area at the 1st–2nd thoracic rib section was traced onto coordinate paper, and the area was determined by measuring the traced region.

### 2.5. Sample Collection

Slaughter was conducted on the experimental farm, without long-distance transport to an off-site slaughter facility. Pigs were fasted for 12 h before slaughter and had access to water during the 4 h lairage period. Both groups followed the same pre-slaughter handling procedures. Before slaughter, blood was collected from the anterior vena cava of the eight selected pigs per group into tubes without anticoagulant. After clotting, samples were centrifuged at 3500× *g* for 10 min at 4 °C, and serum aliquots were stored at −20 °C. Immediately after exsanguination, tissue from the reported last 5th–6th thoracic-rib region was collected for transcriptomic, metabolomic, and RT-qPCR analyses, snap-frozen in liquid nitrogen, and stored at −80 °C. At 45 min post-mortem, samples from the reported 1st–2nd lumbar region were used for early meat color measurement, after which carcasses were chilled at 4 °C. At 24 h post-mortem, samples were collected from the reported 3rd–5th lumbar region for chemical composition, the last 3rd–4th thoracic-rib region for pH measurement, the last 5th–6th thoracic-rib region for western blotting and immunofluorescence, and the last 7th–9th thoracic-rib region for shear-force measurement. Separate portions were allocated to accommodate rapid freezing for molecular analyses, surface and probe measurements, and the preparation of intact muscle blocks for physical and chemical assays. The sampling site for each assay was identical in DLY and TFB pigs, and measurements were matched by animal ID.

### 2.6. Serum Lipid Analysis

Serum triglyceride and total cholesterol concentrations were determined using commercial assay kits (catalogue nos. A110-1-1 and A111-1-1, respectively; Nanjing Jiancheng Bioengineering Institute, Nanjing, China) on an automatic biochemical analyzer (Model 3100; Hitachi High-Technologies Corporation, Tokyo, Japan). Triglyceride was assayed by the glycerol-3-phosphate oxidase–peroxidase (GPO-PAP) method and total cholesterol by the cholesterol oxidase–peroxidase (COD-PAP) method, both according to the manufacturers’ instructions. All samples were analyzed within a single run.

### 2.7. pH Value

LT muscle pH was measured at 45 min post-mortem and after 24 h at 4 °C using a pH STAR meter (SFK-Technology, Copenhagen, Denmark) with automatic temperature compensation, calibrated with pH 4.0 and 7.0 buffers. Five readings per sample were averaged for analysis.

### 2.8. Meat Color Measurement

Early post-mortem meat color was measured on freshly cut LT surfaces at 45 min, without a blooming period [21,22], using a Minolta CR-400 colorimeter (Konica Minolta, Sakai, Japan; D65 illuminant, 2° observer, 5 mm aperture). CIE L*, a*, and b* values were averaged across five locations per sample, avoiding visible fat and connective tissue. The color assessment describes the early post-mortem muscle surface at 45 min. Color after 24 h chilling was not measured.

### 2.9. Drip Loss

Drip loss was measured by suspension [23]. LT samples (approximately 2.0 cm thick, 80–100 g) were surface-blotted, weighed (W0), and suspended in plastic bags containing ambient air at 4 °C for 24 h. Samples were then blotted with filter paper and reweighed (W1). Drip loss (%) = (W0 − W1)/W0 × 100.

### 2.10. Cooking Loss

Trimmed samples (approximately 2.0 cm thick and 5.0 × 5.0 cm) were surface-blotted and weighed (W3), then cooked in a single batch in an 80 °C water bath until the core reached 70 °C. Samples were cooled to room temperature, blotted with filter paper, and reweighed (W4). Cooking loss (%) = (W3 − W4)/W3 × 100.

### 2.11. Shear Force Measurement

Muscle samples were vacuum-sealed in heat-resistant bags and cooked in a 75–80 °C water bath until the core reached 70 °C, which was maintained for 30 min. Samples were cooled in running water to room temperature. Four cores per sample (1.27 cm diameter, approximately 2.0 cm length) were cut parallel to the fibers and sheared perpendicular to them using a Warner–Bratzler texture analyzer (TA. XT Plus, Stable Micro Systems, Godalming, UK). Mean peak force across the four cores was expressed in Newtons (N).

### 2.12. Intramuscular Fat (IMF) Measurement

IMF was measured by Soxhlet extraction. LT samples were trimmed of visible fat and fascia, minced, freeze-dried for 48 h (FUD-1110, Tokyo Rikakikai Co., Ltd., Tokyo, Japan), and dried at 65 °C to constant weight. Ground material was passed through a 1.0 mm sieve; approximately 2.0 g was placed in a cellulose thimble and extracted with petroleum ether for 8 h. After solvent recovery, extraction flasks were dried at 105 °C to constant weight. Extracted fat was expressed as a percentage of sample weight on a fresh-weight basis.

### 2.13. Transcriptome Analysis

RNA was extracted using TRIzol from LT tissue collected at slaughter from six pigs per group, one per pen. Quality was assessed by spectrophotometry and an Agilent 2100 Bioanalyzer (Agilent Technologies, Santa Clara, CA, USA), requiring OD260/280 of 1.8–2.2, OD260/230 ≥ 2.0, RIN ≥ 6.5, 28S:18S ≥ 1.0, and RNA input ≥ 1 μg. Stranded libraries with approximately 300 bp inserts underwent 150 bp paired-end sequencing on an Illumina NovaSeq 6000 (Illumina, San Diego, CA, USA). Reads were filtered using fastp, aligned to *Sus scrofa* 11.1 with Ensembl annotation using HISAT2, and quantified as expected counts and TPM using RSEM (https://deweylab.github.io/RSEM/, accessed on 28 October 2026). DESeq2 results defined DEGs by |log2 fold change| ≥ log2(1.5) and BH-adjusted *p* ≤ 0.05.

KEGG GSEA used a separate limma analysis of log2(TPM + 1), retaining genes with TPM > 0.5 in at least six samples. Duplicate symbols were represented by the highest mean expression. The DLY-versus-TFB model used robust empirical Bayes moderation with a mean–variance trend. Moderated *t* statistics were mapped to Entrez IDs using org.Ss.eg.db, retaining the largest absolute *t* for duplicate IDs. Positive and negative NES indicated DLY and TFB enrichment, respectively; significance required BH-FDR < 0.05. PCA used standardized, filtered log-transformed expression; heatmaps, used row z-scores and the same expression filter. Gene labels, including *ATP6* and *ND6*, followed the supplied annotation.

### 2.14. Metabolomic Analysis

LT samples from six pigs per group were collected at slaughter, immediately frozen in liquid nitrogen and matched by animal ID. Tissue (100 mg) was extracted in 800 μL methanol/water (4:1, *v*/*v*) containing internal standards, including L-2-chlorophenylalanine (0.02 mg/mL). Samples were homogenized at −10 °C and 50 Hz for 6 min, sonicated at 5 °C and 40 kHz for 30 min, incubated at −20 °C for 30 min, and centrifuged at 13,000× *g* for 15 min at 4 °C. Supernatants were analyzed using UHPLC–Orbitrap Exploris 480 (Thermo Fisher Scientific, Bremen, Germany), with an HSS T3 column (100 × 2.1 mm, 1.8 μm) at 40 °C. Positive- and negative-ion acquisition covered *m*/*z* 70–1050, with periodic pooled QC injections.

Progenesis QI performed peak detection, alignment, integration, and normalization. Features with QC RSD > 30% were excluded; remaining abundances were normalized to total ion intensity and log10-transformed. HMDB, METLIN, and Majorbio annotations used accurate mass, isotope patterns, and MS/MS spectra, and were reported as putative MSI levels 2 or 3 without authentic standard confirmation.

PCA used centered, variance-scaled abundances. PLS-DA in ropls used two predictive components, standard scaling, six-fold cross-validation, and 200 permutations (seed 20261003). Student’s *t*-tests provided the primary comparison consistent with the exported results; Welch’s *t*-tests and Mann–Whitney *U* tests assessed sensitivity. Each analysis applied BH correction across all detected features. Candidates required VIP > 1 and unadjusted *p* < 0.05; corrected differences required BH-FDR < 0.05.

Pathway enrichment used deduplicated candidate names and 275 detected KEGG-mapped metabolites as background. One-sided Fisher tests evaluated 122 pathways with at least three background members, with BH correction across all pathways. Sensitivity analysis used Welch *p* < 0.05 and VIP > 1 with identical background and correction criteria (Supplementary Table S5b). Measured contrasts were overlaid on reference histidine-related reactions, using row-standardized profiles and log2 geometric-mean abundance ratios; related annotations without verified reaction assignments are shown separately.

### 2.15. RT-qPCR

Total RNA was extracted using RNA Isolater Total RNA Extraction Reagent (Vazyme, Nanjing, China) and reverse-transcribed into cDNA with HiScript II Q RT Supermix (Vazyme). Quantitative real-time PCR was performed using ChamQ SYBR Color qPCR Master Mix (Vazyme) on a 7900HT Real-Time PCR System (Applied Biosystems, Foster City, CA, USA). Reactions included no-template controls and were performed in technical triplicate. A single melting-curve peak was required for each assay. *GAPDH* served as the internal reference gene, and relative expression was calculated using the 2−ΔΔCt method. Primer sequences are provided in Supplementary Table S2. Target genes included *MyHC I*, *MyHC IIa*, *MyHC IIx*, and *MyHC IIb*.

### 2.16. Western Blotting

Frozen Longissimus thoracis samples were homogenized in RIPA buffer containing protease and phosphatase inhibitors and centrifuged at 12,000× *g* for 15 min at 4 °C. Protein concentrations were determined using a BCA assay. Equal amounts of protein were separated by SDS-PAGE, transferred to PVDF membranes, blocked with 5% nonfat milk, and incubated with primary antibodies overnight at 4 °C, followed by HRP-conjugated secondary antibodies. Bands were visualized by enhanced chemiluminescence using a ChemiDoc system (Bio-Rad, Tokyo, Japan) and quantified in Image Lab (https://www.bio-rad.com/en-us/product/image-lab-software?ID=KRE6P5E8Z, accessed on 27 October 2026). Phosphorylated proteins were expressed relative to their corresponding total proteins; other targets were normalized to GAPDH or β-actin and expressed relative to the DLY group [22,24]. Primary antibodies included anti-mTOR (Cell Signaling Technology, Danvers, MA, USA, 2972), anti-phospho-mTOR Ser2448 (3018), anti-AKT (4691), anti-phospho-AKT Ser473 (4060), anti-S6K1 (9202), anti-phospho-S6K1 Thr389 (9205), anti-4E-BP1 (9452), anti-phospho-4E-BP1 Thr37/46 (2855), anti-SIRT1 (Proteintech, Rosemont, IL, USA, 13161-1-AP), anti-AMPKα (Cell Signaling Technology, 5831), anti-phospho-AMPKα Thr172 (2535), anti-fast MyHC (Servicebio, Wuhan, China, GB112130), anti-slow MyHC (Servicebio, GB150144), anti-GAPDH (Servicebio, GB15004), and anti-β-actin (Servicebio, GB15003). For all western-blot targets, protein extracts from six pigs per group were combined in pairs using equal protein amounts from each animal. Within each group, pigs from different pens were paired (1–2, 3–4, and 5–6) to form three independent pools for western blotting.

### 2.17. Immunofluorescence of LT Muscle

LT samples were fixed for 24 h and paraffin-embedded. Sections (4 μm) were deparaffinized, rehydrated, subjected to antigen retrieval, and blocked before overnight incubation at 4 °C with anti-slow MyHC (Abcam, Waltham, MA, USA, ab11083) and anti-fast MyHC (Abcam, ab91506). FITC-conjugated goat anti-mouse and Cy3-conjugated goat anti-rabbit secondary antibodies were applied for 50 min at room temperature in the dark. Sections were counterstained with DAPI, mounted in anti-fade medium, and imaged using a Nikon fluorescence microscope with a 20× objective (Tokyo, Japan). Slow- and fast-MyHC-positive staining areas were quantified as percentages using ImageJ version 1.54. Two sections per pig were evaluated, with five microscopic fields selected from each section (10 fields per pig). Fields were selected from intact, undamaged tissue regions. Positive-staining areas were identified using automatic thresholding in ImageJ, with uniform analysis criteria applied across both groups. Image quantification was performed by an operator blinded to production group.

### 2.18. Statistical Analysis

Terminal carcass, meat quality, and serum lipid outcomes were compared using two-sided Welch’s *t*-tests (eight pigs per group, one per pen). The model was Yi = μ + βGroupi + εi, with production group as the fixed effect and no body weight adjustment. Growth performance comparisons used two-sided Welch’s *t*-tests, with eight independent pens per group for initial and final body weight, ADG, ADFI, and F/G. All western blot analyses used three independent protein pools per group. Results are presented as mean ± SEM, except for initial individual body weights in Section 2.2 (mean ± SD). Exact *p* values are reported unless *p* < 0.001. Between-group mean differences (TFB minus DLY) and Welch–Satterthwaite 95% confidence intervals are provided in Supplementary Table S7. Omics tests and BH correction are described in Section 2.13 and Section 2.14. Group-adjusted partial Spearman correlations were calculated for 12 matched animals, with BH correction across all 180 feature–trait tests. Coefficients, unadjusted *p* values, and adjusted *q* values are reported together; *p* < 0.05 denotes nominal significance. Non-omics analyses used SPSS 20.0, and correlations were checked in Python version 3.12.14 and R version 4.6.1 ppcor [25]. SEMs were calculated separately for each group as the within-group sample SD divided by the square root of the number of independent experimental units.

## 3. Results

### 3.1. Growth Performance

Growth performance is summarized in Table 1. DLY pigs had higher initial body weight (98.22 ± 1.61 vs. 72.76 ± 0.43 kg), final body weight (143.11 ± 1.93 vs. 102.12 ± 0.39 kg), ADG (1.069 ± 0.020 vs. 0.699 ± 0.004 kg/d), and ADFI (3.558 ± 0.067 vs. 2.436 ± 0.026 kg/d) than TFB pigs (all *p* < 0.001). F/G was lower in DLY than TFB (3.332 ± 0.057 vs. 3.486 ± 0.033; *p* = 0.0388).

### 3.2. Carcass Characteristics

As shown in Table 2, at 195 days of age, TFB pigs exhibited significantly lower carcass weight, carcass length, and loin eye area compared with DLY pigs. In contrast, backfat thickness at the first rib and last lumbar vertebra, as well as average backfat thickness, were significantly higher in TFB pigs, while the difference at the last rib did not reach statistical significance (*p* = 0.057). TFB pigs exhibited a lower dressing percentage than DLY pigs (72.54 ± 0.40 vs. 76.76 ± 0.55%, *p* < 0.001).

### 3.3. Meat Quality

**Figure 1:**
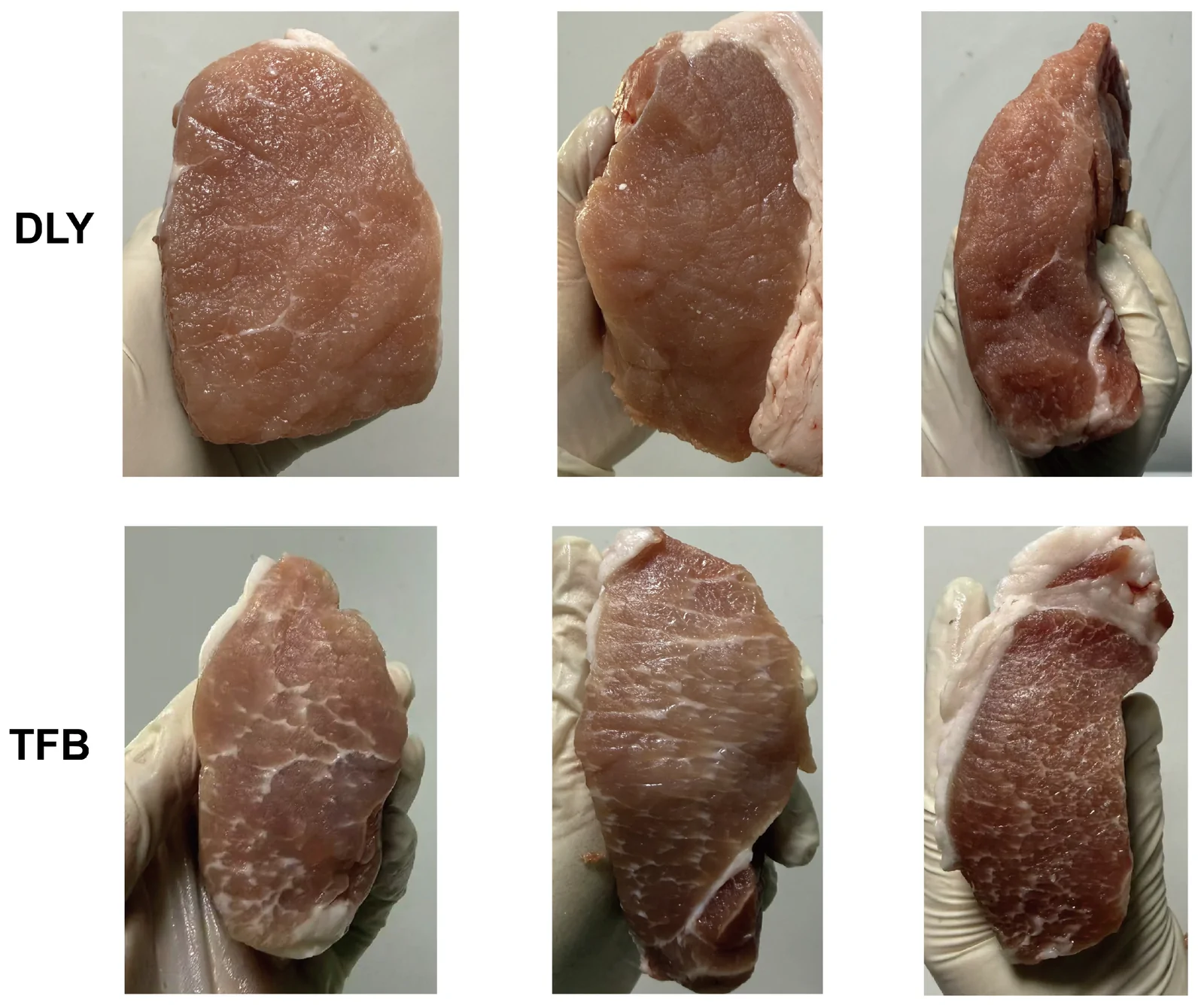
Representative LT cross-sections showing gross appearance and marbling. Three samples per group are shown, DLY, upper row; TFB, lower row. Images illustrate appearance and were not quantified.

TFB pigs had lower drip loss (2.66 ± 0.33 vs. 4.12 ± 0.27%; *p* = 0.004), cooking loss (27.88 ± 1.59 vs. 32.44 ± 0.61%; *p* = 0.025), and shear force (43.50 ± 3.10 vs. 55.46 ± 1.61 N; *p* = 0.006) than DLY pigs (Table 3). Intramuscular fat was higher in TFB (3.42 ± 0.16 vs. 2.18 ± 0.07%; *p* < 0.001). Neither pH45min nor the 45-min color parameters differed significantly between groups; pH24h was higher in TFB (5.74 ± 0.02 vs. 5.69 ± 0.01; *p* = 0.0486). Between-group mean differences and 95% confidence intervals are provided in Supplementary Table S7. Figure 1 provides representative gross images.

### 3.4. Serum Lipid Profiles

Fasting serum triglyceride and total cholesterol concentrations were higher in DLY pigs than in TFB pigs (0.523 ± 0.029 vs. 0.327 ± 0.028 mmol/L, *p* < 0.001; and 3.206 ± 0.084 vs. 2.643 ± 0.110 mmol/L, *p* = 0.001, respectively; Table 4). This contrasted with the greater intramuscular fat content and backfat thickness observed in TFB pigs.

### 3.5. Muscle Fiber Type Composition

**Figure 2:**
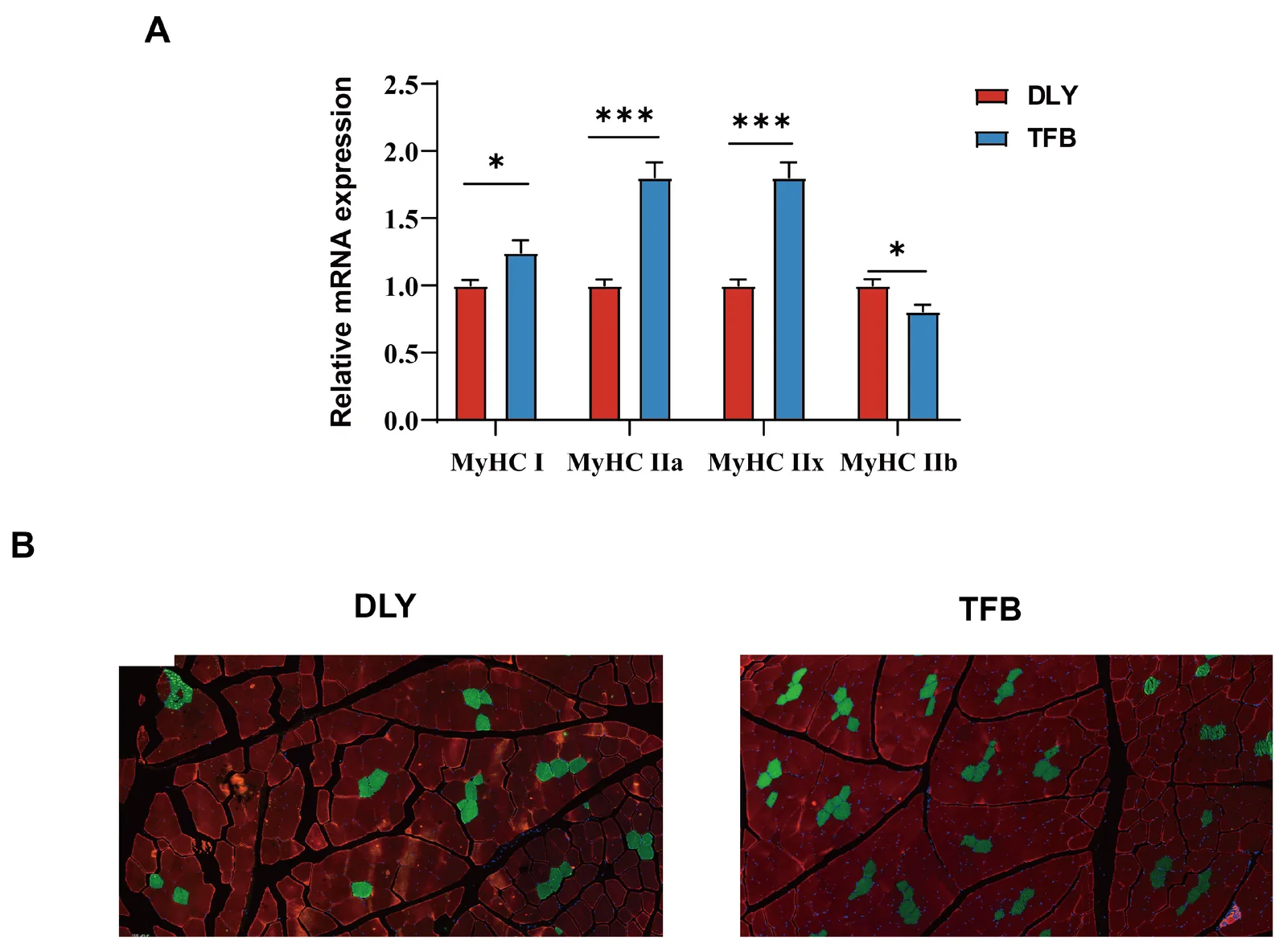
Muscle fiber characteristics. (**A**) Relative *MyHC I*, IIa, IIx and IIb mRNA abundance. (**B**) Representative fast MyHC (red), slow MyHC (green), and nuclear (blue) staining, with positive area quantification. Bars represent group means, and error bars indicate SEM;; *n* = 8 pigs per group; two-sided Welch’s *t*-tests. * *p* < 0.05; *** *p* < 0.001.

We first examined muscle fiber characteristics using MyHC isoform expression and immunofluorescence (Figure 2). TFB pigs showed higher *MyHC I*, *MyHC IIa*, and *MyHC IIx* mRNA abundance and lower *MyHC IIb* abundance than DLY pigs (*p* < 0.05; Figure 2A). Immunofluorescence showed a greater slow MyHC–positive staining area in TFB muscle (Figure 2B).

### 3.6. Targeted Muscle Protein Profiles

**Figure 3:**
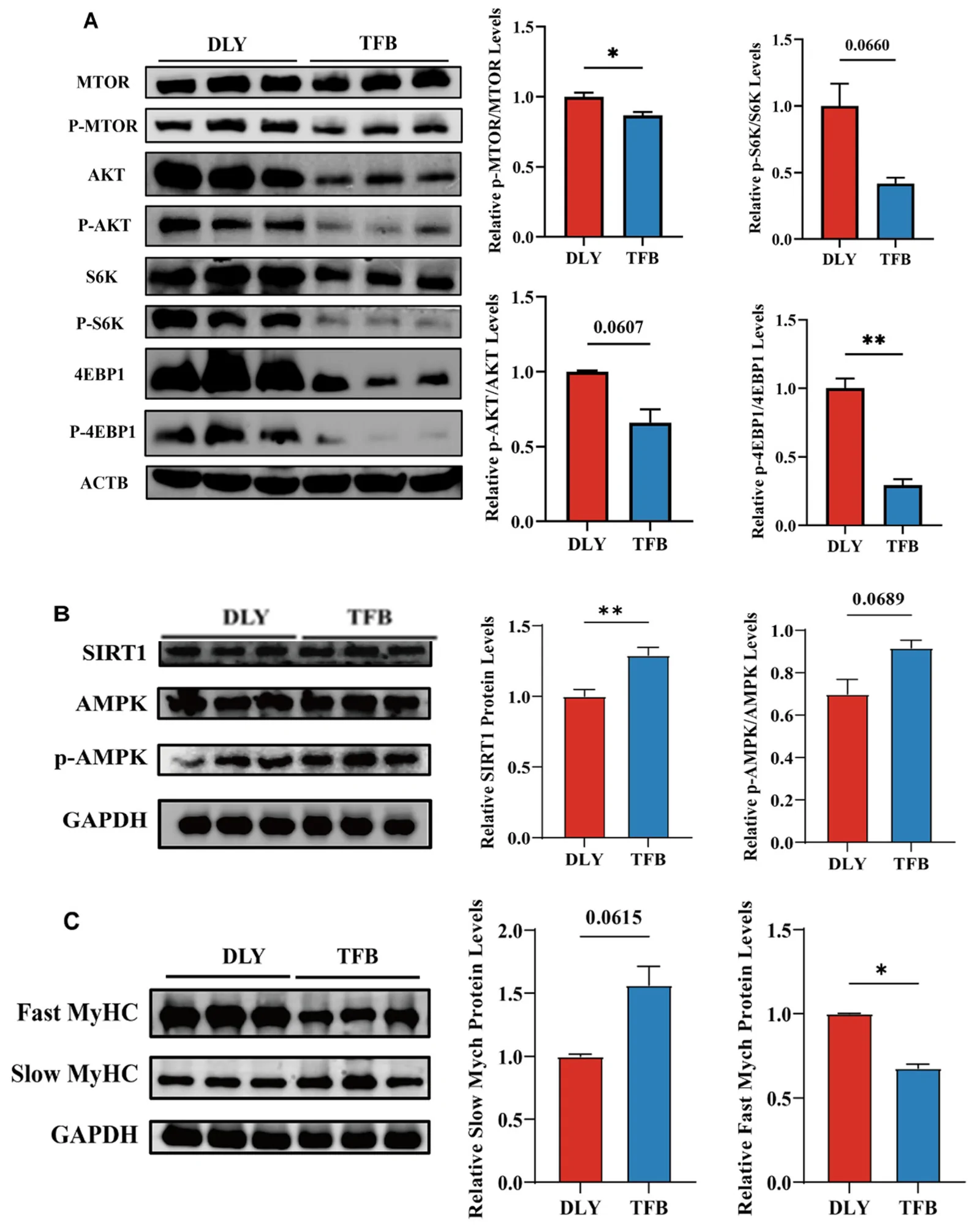
Muscle protein profiles. (**A**) Immunoblots and phosphorylation-to-total protein ratios for mTOR, AKT, S6K, and 4EBP1. (**B**) SIRT1 abundance and p-AMPK/AMPK. (**C**) Slow and fast MyHC abundance. Loading controls, ACTB in (**A**); GAPDH in (**B**,**C**). Mean ± SEM; *n* = 3 independent equal-protein pools per group, each formed from 2 pigs. Two-sided Welch’s *t*-tests; * *p* < 0.05; ** *p* < 0.01. DLY, red; TFB, blue.

Targeted western blotting was then used to determine whether the muscle fiber phenotype was accompanied by differences in anabolic and energy sensing signaling (Figure 3). The mTOR, AKT, S6K, and 4EBP1 proteins and their phosphorylated forms were quantified as indicators of the mTOR-AKT anabolic signaling axis (Figure 3A).

The p-mTOR/mTOR and p-4EBP1/4EBP1 ratios were higher in DLY than TFB muscle (*p* < 0.05; Figure 3A). Differences in p-AKT/AKT and p-S6K/S6K did not reach significance (*p* = 0.0607 and 0.0660, respectively).

SIRT1 abundance was higher in TFB muscle, whereas the p-AMPK/AMPK difference did not reach significance (*p* = 0.0689; Figure 3B). Fast MyHC abundance was lower in TFB (*p* < 0.05), while the slow MyHC protein difference did not reach significance (*p* = 0.0615; Figure 3C). These measurements used three independent protein pools per group, each containing equal protein amounts from two pigs.

### 3.7. Skeletal Muscle Transcriptomic Profiles and Pathway Enrichment

**Figure 4:**
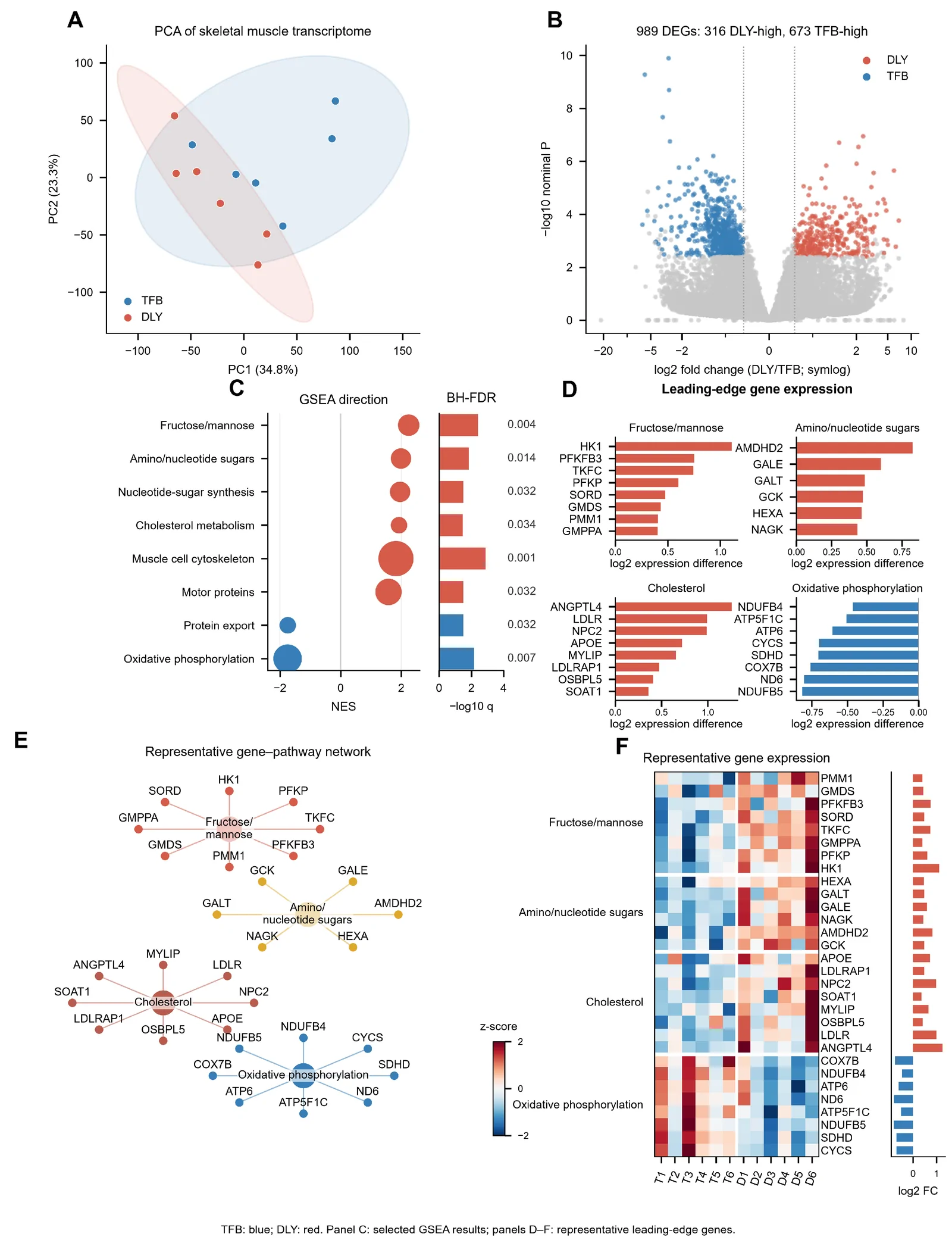
Muscle transcriptomic profiles. (**A**) PCA; ellipses, 95% covariance contours. (**B**) DESeq2 contrasts at |log2 fold change| ≥ log2(1.5) and BH-FDR ≤ 0.05, 316 DLY-high and 673 TFB-high genes; remaining genes are gray. The horizontal axis uses a symmetric log scale. (**C**) Eight significant GSEA pathways selected by functional relevance, rather than adjusted *p* ranking; selected results, Supplementary Table S5a. Positive/negative NES denotes DLY/TFB enrichment. Bubble area, leading-edge gene count; bars, adjusted *p*. (**D**) Limma log2 expression differences for representative leading-edge genes. (**E**) Gene–pathway links. (**F**) Row-standardized expression and corresponding limma contrasts. T1–T6, TFB; D1–D6, DLY; *n* = 6 pigs per group.

Muscle transcriptomic PCA showed differences in sample distribution, with PC1 and PC2 explaining 34.8% and 23.3% of variance, respectively (Figure 4A). Applying |log2 fold change| ≥ log2(1.5) and BH-FDR ≤ 0.05 to the DESeq2 export identified 989 genes, including 673 higher in TFB and 316 higher in DLY (Figure 4B; Supplementary Table S3). The separate limma-based GSEA retained 35 pathways at BH-FDR < 0.05; eight selected pathways are shown in Figure 4C. DLY enrichment included fructose and mannose metabolism (*q* = 0.004), amino sugar and nucleotide sugar metabolism (*q* = 0.014), biosynthesis of nucleotide sugars (*q* = 0.032), cholesterol metabolism (*q* = 0.034), cytoskeleton in muscle cells (*q* = 0.001), and motor proteins (NES = 1.579, *q* = 0.032). Oxidative phosphorylation (NES = −1.749, *q* = 0.007) and protein export (NES = −1.743, *q* = 0.032) were enriched in TFB. Representative leading-edge gene expression differences, gene–pathway links, and sample-level expression profiles summarize the carbohydrate-, cholesterol-, and mitochondrial-expression patterns (Figure 4D–F).

### 3.8. Skeletal Muscle Metabolomic Profiles and Pathway Enrichment

**Figure 5:**
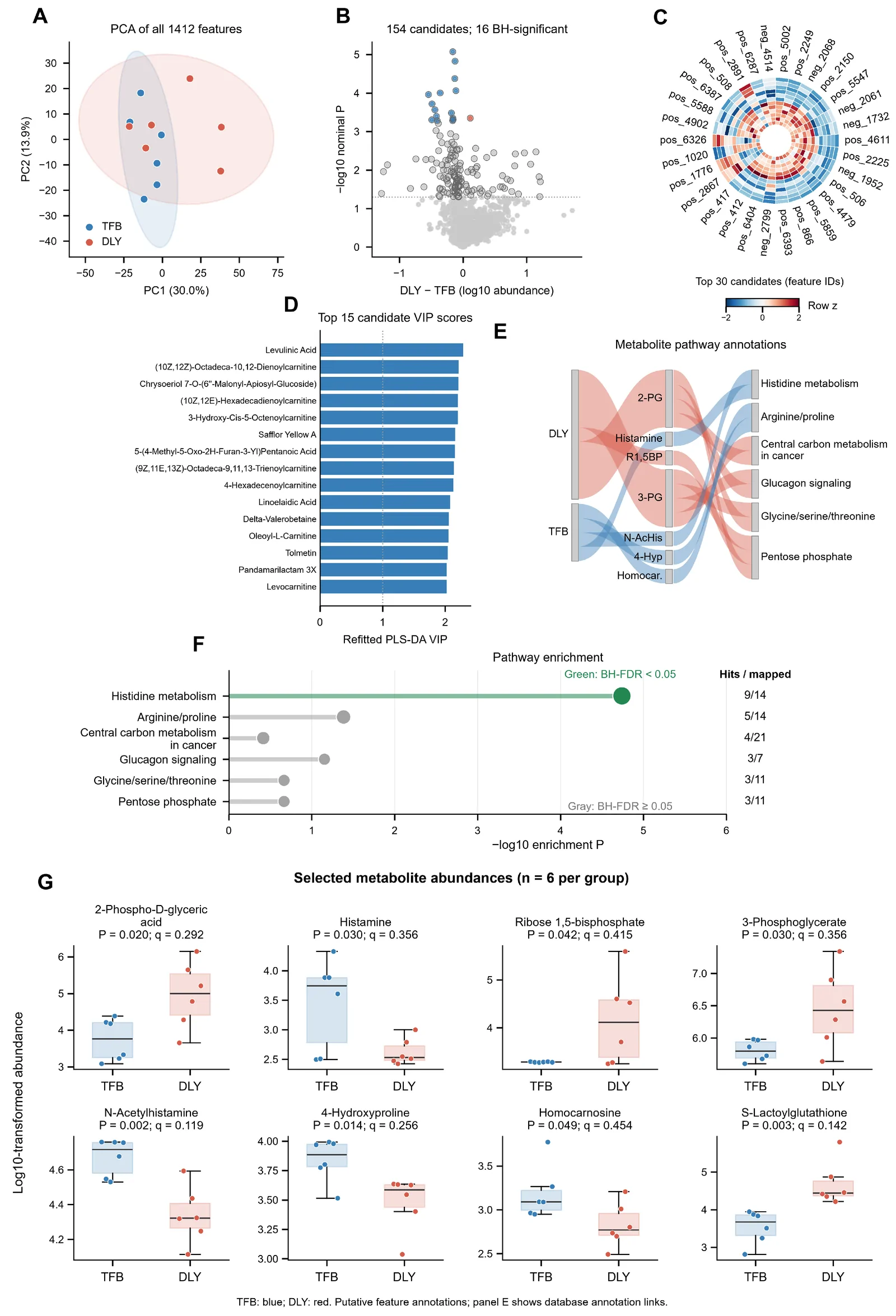
Muscle metabolomic profiles. (**A**) PCA; ellipses, 95% covariance contours. (**B**) Circles mark 154 candidates (VIP > 1; Student *p* < 0.05); red/blue fills mark 16 BH-significant features. The horizontal axis shows DLY-minus-TFB log10 abundance. (**C**) Row-standardized profiles of the top 30 candidate VIP features; inner/outer six rings, TFB/DLY. (**D**) Top 15 VIP scores. (**E**) Count-weighted metabolite–pathway links. (**F**) Measured-background enrichment, lines, −log10 *p*; point area, candidate hits; green/gray, BH-FDR < 0.05/≥0.05. The right column gives candidate/background counts; histidine-pathway results, Supplementary Table S5b. (**G**) Selected log10 abundance profiles. Points represent individual animals; boxes show the median and interquartile range. Whiskers indicate the range of observations within 1.5 times the interquartile range below the first quartile and above the third quartile; caps mark the whisker endpoints.Annotations are putative. *n* = 6 pigs per group; T, TFB (blue); D, DLY (red); 2-PG, 2-phospho-D-glyceric acid; 3-PG, 3-phosphoglycerate; R1,5BP, ribose 1,5-bisphosphate; N-AcHis, N-acetylhistamine; 4-Hyp, 4-hydroxyproline; Homocar., homocarnosine.

PCA of all 1412 detected metabolomic features showed a contrast between DLY and TFB, with PC1 and PC2 explaining 30.0% and 13.9% of variance (Figure 5A). The refitted two-component PLS-DA model yielded R2Y = 0.946 and cross-validated Q2 = 0.655; the permutation *p* values were 0.325 for R2Y and 0.030 for Q2. Student *t*-tests and VIP > 1 identified 154 candidate features, including 40 higher in DLY and 114 higher in TFB. Across the complete feature set, 16 met BH-FDR < 0.05 in the primary Student’s *t*-test analysis, with 15 higher in TFB and one in DLY (Figure 5B; Supplementary Table S4). These included the putative annotations 1-methylhistidine and N-acetyl-1-methylhistidine, five acylcarnitines, and levocarnitine. Their individual feature results are distinct from those of the selected histamine, N-acetylhistamine, and homocarnosine candidates and from the pathway-level histidine enrichment test. Welch testing retained five BH-significant features on the same log10 abundance scale, all within the primary 16-feature set; Mann–Whitney testing retained none, indicating sensitivity of feature-level findings to the comparison method. Among selected putative annotations, 2-phospho-D-glyceric acid, 3-phosphoglycerate, ribose 1,5-bisphosphate, and S-lactoylglutathione were higher in DLY, whereas histamine, N-acetylhistamine, homocarnosine, and 4-hydroxyproline were higher in TFB (Figure 5G). Histamine, N-acetylhistamine, homocarnosine, and S-lactoylglutathione had nominal *p* values of 0.0302, 0.0019, 0.0488, and 0.0034, respectively; their BH-FDR values were 0.356, 0.119, 0.454, and 0.142. Measured-background enrichment identified histidine metabolism (9 of 14 mapped background members; BH-FDR = 0.0022) as the pathway retained after correction across 122 tests (Figure 5F; Supplementary Table S5b). The other displayed pathways had BH-FDR ≥ 0.05.

### 3.9. Cross-Omics Comparison and Associations with Meat Quality Traits

**Figure 6:**
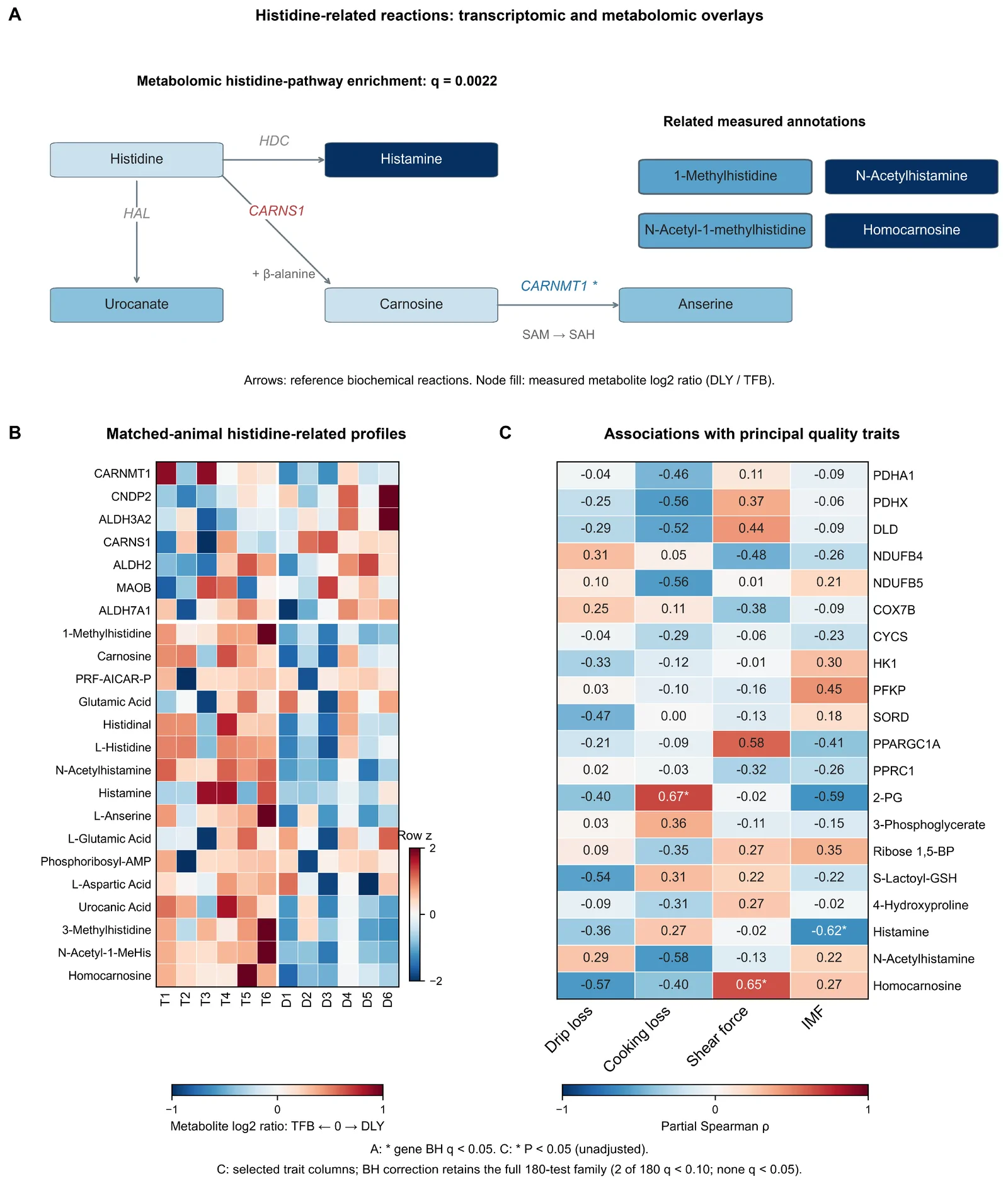
Histidine-related profiles and meat-quality associations. (**A**) Reference reactions with measured expression and putative abundance contrasts. Blue/red, higher in TFB/DLY; gray gene labels, below the expression filter; gene asterisks, adjusted *p* < 0.05. Metabolite fill shows log_2_ abundance ratios (DLY/TFB). (**B**) Row-standardized profiles of seven genes and 16 mapped or related metabolite annotations; T1–T6, TFB; D1–D6, DLY. (**C**) Group-adjusted partial Spearman correlations with four meat-quality traits in 12 matched animals. Cells show coefficients; * indicates unadjusted *p* < 0.05. BH correction covers all 180 tests; two reached *q* < 0.10 and none reached *q* < 0.05. Selected associations are listed in Supplementary Table S6. SAM, S-adenosylmethionine; SAH, S-adenosylhomocysteine.

Transcriptomic oxidative phosphorylation enrichment in TFB and metabolomic histidine pathway enrichment were considered alongside the muscle fiber and meat quality profiles. Figure 6A maps measured molecular contrasts onto reference histidine-related reactions. *CARNMT1* expression was higher in TFB (log2 fold change, DLY/TFB = −0.977; BH-adjusted *p* = 0.030). Carnosine and anserine abundances were also higher in TFB in the primary comparisons (*p* = 0.0256 and 0.0087). These results place the expression and abundance contrasts within the carnosine–anserine branch. Figure 6B shows the matched-animal profiles of seven histidine-related genes meeting the expression filter and 16 mapped or related metabolite annotations, including members without significant group differences. Group-adjusted partial Spearman analysis in 12 matched animals identified 17 associations with unadjusted *p* < 0.05 among 180 tests. Two reached BH *q* < 0.10, N-acetylhistamine with pH24h (ρ = 0.867, *p* = 0.000541, *q* = 0.0562) and *CYCS* with a* at 45 min (ρ = 0.863, *p* = 0.000624, *q* = 0.0562); none reached *q* < 0.05. Figure 6C displays the four principal meat quality traits. Exploratory associations were observed for homocarnosine and shear force (ρ = 0.646, *p* = 0.0319, *q* = 0.429), 2-phospho-D-glyceric acid and cooking loss (ρ = 0.671, *p* = 0.0237, *q* = 0.429), and histamine and IMF (ρ = −0.620, *p* = 0.0420, *q* = 0.429). Homocarnosine–drip loss and N-acetylhistamine–cooking loss had *p* values of 0.0663 and 0.0640, respectively. Selected associations are reported in Supplementary Table S6.

## 4. Discussion

The common-age comparison revealed contrasting patterns of growth, carcass development, and meat quality in DLY and TFB pigs. During the 42-day finishing period, DLY had higher ADG and ADFI and lower F/G, together with greater carcass weight, carcass length, and loin eye area. TFB had greater backfat thickness and IMF. These findings agree with comparisons of commercial crossbreds and indigenous or quality-oriented pigs, in which lean growth and fat deposition differ among production types [1,2,3,26,27]. Studies comparing Landrace with Lantang pigs and Yorkshire with Shaziling pigs at matched chronological ages have likewise documented differences in carcass development and muscle characteristics [28,29]. A common-age design therefore provides a relevant framework for examining how DLY and TFB pigs differ at the same stage of the rearing period.

Lower drip loss, cooking loss, and shear force were the principal meat quality features of TFB. These traits describe differences in water-holding capacity, shear force, and IMF content between DLY and TFB. Previous studies of indigenous and commercial pigs have reported variation in muscle composition and meat quality traits [2,3,10]. Wood et al. and Hocquette et al. describe IMF as an important component of meat quality whose deposition depends on genetic background, nutrition, and development [26,27]. The higher IMF content and lower shear force in TFB are consistent with the relationship between muscle composition and meat quality described in these studies. Water loss and shear force also depend on muscle structure and post-mortem protein changes, making muscle fiber characteristics a useful link between the observed phenotypes and molecular profiles.

TFB pigs had lower fasting serum triglyceride and total cholesterol concentrations despite higher IMF content and greater backfat thickness. A comparable pattern was reported by Peng et al., who found higher IMF and backfat thickness together with lower plasma triglyceride concentrations in weight-matched Laiwu pigs than in Yorkshire pigs [30]. Tracer studies in pigs have separately quantified hepatic VLDL-triglyceride secretion and circulating VLDL-triglyceride turnover [31], illustrating the distinction between circulating lipid concentrations and metabolic rates. The present serum and tissue measurements therefore describe differences in circulating lipid concentrations and accumulated fat stores. Altered lipid partitioning is a possible explanation, but testing it would require direct measurements of lipid synthesis, uptake, and turnover.

The oxidative muscle fiber profile of TFB provides a biological context for its lower water losses. Higher MyHC I and IIa transcript abundance and a greater slow MyHC–positive area were accompanied by oxidative phosphorylation enrichment. Ryu and Kim found that type I and IIa fiber area percentages were negatively associated with drip loss, whereas type IIb was positively associated [32]. Their related work connected rapid post-mortem metabolism with protein denaturation [33]. Breed comparisons and reviews have also emphasized the relationship between fiber composition, muscle metabolism, and pork quality [8,9,10]. The agreement between the fiber markers, transcriptomic enrichment, and water-loss phenotype in TFB is thus consistent with established porcine muscle biology. Differences in CYCS, SDHD, NDUFB4, NDUFB5, COX7B, and ATP5F1C expression further implicate mitochondrial electron transport and ATP synthesis.

The mTOR and 4EBP1 phosphorylation results may be related to differences in muscle growth. DLY showed higher p-mTOR/mTOR and p-4EBP1/4EBP1 ratios alongside its larger carcass and loin eye area. mTOR signaling regulates skeletal muscle growth and translational control [34], making these phosphorylation differences relevant to the growth phenotype. Liu et al. reported lower phosphorylation ratios of several mTOR-pathway proteins in Bama mini-pigs than in Landrace pigs, together with differences in muscle amino-acid composition [35]. Their findings help explain why growth-related signaling and individual meat quality traits can follow different patterns across pig types. In this comparison, the mTOR and 4EBP1 profiles correspond to the DLY growth phenotype, while the TFB fiber and composition profiles correspond to lower water losses and shear force. Higher SIRT1 abundance in TFB adds an energy regulatory feature, consistent with the role of SIRT1 in skeletal muscle metabolic adaptation [36].

Pathway analysis extended these muscle characteristics into distinct transcriptional profiles. Oxidative phosphorylation in TFB complemented the fiber marker findings, whereas carbohydrate- and cholesterol-related pathways were enriched in DLY. Carbohydrate metabolism connects substrate availability with muscle energy supply, and cholesterol metabolism contributes to membrane composition and cellular lipid handling. Enrichment of motor protein and cytoskeletal pathways in DLY highlighted muscle structural organization alongside its greater loin eye area. Previous porcine metabolomic and integrative studies have also linked energy metabolism profiles with drip loss and other pork quality traits [37,38]. These enriched pathways suggest differences in muscle energy metabolism and structural organization between DLY and TFB.

Histidine metabolism emerged as a second molecular theme. Its pathway enrichment in TFB was accompanied by higher *CARNMT1* expression and higher measured carnosine and anserine abundances, placing the cross-omics comparison within the carnosine methylation branch. Carnosine has buffering and antioxidant properties relevant to the biochemical changes accompanying conversion of muscle to meat. D’Astous-Pagé et al. associated higher carnosine content in porcine longissimus muscle with higher pH24h and greater water-holding capacity [39]. Ma et al. also reported effects of dietary carnosine on antioxidant capacity and meat quality in finishing pigs [40]. These studies provide a specific biological basis for examining histidine-containing dipeptides alongside the lower water losses in TFB. Carnosine is often more abundant in glycolytic than oxidative muscle [39], indicating that fiber composition and dipeptide metabolism capture different aspects of the muscle phenotype. The dipeptide profiles and histidine-pathway enrichment provide a biochemical context for studying muscle buffering and water retention. The exploratory feature trait associations require independent validation and do not establish specific biological relationships. The DLY-associated S-lactoylglutathione contrast adds a further metabolic feature through its role in the glyoxalase system and carbonyl metabolism [41].

Overall, the study brings together the major meat quality differences with oxidative fiber characteristics, mitochondrial expression and histidine-related metabolism. The common-age comparison includes variation in body size and physiological maturity, and production type was confounded with commercial source. Metabolite identities remain putative. The histidine-related profiles identify biochemical candidates for further investigation alongside the muscle fiber findings. These considerations define the scope for subsequent work. Targeted quantification of histidine-containing dipeptides, measurements of muscle buffering and oxidative processes, and validation in independent animals would directly evaluate the relationships highlighted here.

## 5. Conclusions

Under the common-age conditions examined, TFB pigs had lower drip loss, cooking loss, and shear force, and higher intramuscular fat content than DLY pigs. These phenotypes accompanied oxidative fiber markers and transcriptomic enrichment of oxidative phosphorylation. DLY showed contrasting carbohydrate-, cholesterol-, and motor-protein expression modules. Metabolomic histidine-pathway enrichment and higher *CARNMT1* expression in TFB placed histidine-related metabolism, including the carnosine–anserine branch, within the cross-omics comparison. The combined findings identify mitochondrial and histidine-related features associated with pork-quality variation in the sampled DLY and TFB pigs. Targeted metabolite measurements and functional studies can examine their contributions.

## Figures and Tables

**Table 1:** Comparison of growth performance between DLY and TFB pigs.

Item	DLY (*n* = 8)	TFB (*n* = 8)	*p*-Value
Initial BW (kg)	98.22 ± 1.61	72.76 ± 0.43	<0.001
Final BW (kg)	143.11 ± 1.93	102.12 ± 0.39	<0.001
ADG (kg/d)	1.069 ± 0.020	0.699 ± 0.004	<0.001
ADFI (kg/d)	3.558 ± 0.067	2.436 ± 0.026	<0.001
F/G	3.332 ± 0.057	3.486 ± 0.033	0.039

Values are presented as mean ± SEM (*n* = 8 pens per group, 3 pigs per pen). The pen was the experimental unit. Groups were compared using two-sided Welch’s *t*-tests. BW, body weight; ADG, average daily gain; ADFI, average daily feed intake; F/G, feed-to-gain ratio. SEMs were calculated separately for each group as SD/√*n*.

**Table 2:** Carcass characteristics of DLY and TFB pigs slaughtered at 195 days of age.

Items	DLY	TFB	*p*-Value
Carcass weight, kg	105.42 ± 1.34	74.08 ± 0.83	<0.001
Dressing percentage, %	76.76 ± 0.55	72.54 ± 0.40	<0.001
Carcass length, cm	112.21 ± 0.83	96.07 ± 1.42	<0.001
Backfat thickness, cm			
First rib	3.98 ± 0.23	5.16 ± 0.24	0.003
Last rib	2.31 ± 0.13	2.96 ± 0.27	0.057
Last lumbar vertebra	1.77 ± 0.15	3.14 ± 0.17	<0.001
Average backfat	2.69 ± 0.13	3.75 ± 0.20	<0.001
Loin-eye area, cm^2^	62.40 ± 5.22	44.86 ± 2.58	0.012

Values are presented as mean ± SEM (*n* = 8 pigs per group, 1 pig sampled per pen). Groups were compared using two-sided Welch’s *t*-tests. Exact *p* values are reported unless *p* < 0.001. DLY, Duroc × (Landrace × Yorkshire); TFB, Tianfu Black; SEM, standard error of the mean. SEMs were calculated separately for each group as SD/√*n*.

**Table 3:** Postmortem physicochemical and quality traits of the longissimus thoracis muscle in DLY and TFB pigs.

Items.	DLY	TFB	*p*-Value
pH_45min_	6.20 ± 0.02	6.17 ± 0.05	0.594
pH_24h_	5.69 ± 0.01	5.74 ± 0.02	0.049
L*_45min_	41.08 ± 0.52	40.08 ± 0.65	0.254
a*_45min_	11.12 ± 0.65	11.91 ± 0.49	0.348
b*_45min_	10.08 ± 0.15	10.76 ± 0.34	0.102
Drip loss, %	4.12 ± 0.27	2.66 ± 0.33	0.004
Cooking loss, %	32.44 ± 0.61	27.88 ± 1.59	0.025
Shear force, N	55.46 ± 1.61	43.50 ± 3.10	0.006
Intramuscular fat, %	2.18 ± 0.07	3.42 ± 0.16	<0.001

Values are presented as mean ± SEM (*n* = 8 pigs per group, 1 pig sampled per pen). Groups were compared using two-sided Welch’s *t*-tests. Exact *p* values are reported unless *p* < 0.001. DLY, Duroc × (Landrace × Yorkshire); TFB, Tianfu Black; SEM, standard error of the mean. SEMs were calculated separately for each group as SD/√*n*. L*, a*, and b* denote CIE lightness, redness and yellowness, respectively; their asterisks are part of the coordinate names.

**Table 4:** Fasting serum triglyceride and total cholesterol concentrations in DLY and TFB pigs.

Items	DLY	TFB	*p*-Value
Triglycerides, mmol/L	0.523 ± 0.029	0.327 ± 0.028	<0.001
Total cholesterol, mmol/L	3.206 ± 0.084	2.643 ± 0.110	0.001

Values are presented as mean ± SEM (*n* = 8 pigs per group, 1 pig sampled per pen). Groups were compared using two-sided Welch’s *t*-tests. Exact *p* values are reported unless *p* < 0.001. DLY, Duroc × (Landrace × Yorkshire); TFB, Tianfu Black; SEM, standard error of the mean. SEMs were calculated separately for each group as SD/√*n*.

## Data Availability

The original contributions presented in this study are included in the article/Supplementary Material. Further inquiries can be directed to the corresponding author.

## Supplementary Materials

The following supporting information can be downloaded at: https://www.mdpi.com/article/10.3390/foods15213932/s1, Table S1: Composition and nutrient levels of the experimental diet (as-fed basis); Table S2: Primer sequences used for RT-qPCR; Table S3: Transcriptomic summary; Table S4: Primary BH-significant metabolites; Table S5a: Selected GSEA results; Table S5b: Histidine-pathway enrichment; Table S6: Exploratory associations with unadjusted *p* < 0.05; Table S7: Mean differences and 95% confidence intervals.
