# Supplementary material for: Multi-Omics Analysis Reveals Differences in Pork Quality and Skeletal Muscle Characteristics Between Tianfu Black and DLY Pigs
Source: Foods. 2026 Nov 2;15(21):3932. doi: 10.3390/foods15213932 (PMC13763469; doi:10.3390/foods15213932)
Supplement: Supplementary file 1 [file foods-15-03932-s001.zip › foods-4566201-supplementary.pdf]

Supplementary Materials

Contrasting pork quality phenotypes and associated skeletal muscle multi-omic profiles in Tianfu Black and commercial crossbred pigs

Supplementary Table S1. Composition and nutrient levels of the experimental diet (as-fed basis).

Item	Content
Corn	57.8
Wheat	20
Wheat bran	12.85
Soybean meal (CP 46.0%)	6.7
Limestone	0.735
Dicalcium phosphate	0.675
NaCl	0.3
L-Lysine HCl (98.0%)	0.355
DL-Methionine (99.0%)	0.0135
L-Threonine (98.5%)	0.117
L-Tryptophan (30.0%)	0.07
Choline chloride (60.0%)	0.1
Mineral premix[1]	0.15
Vitamin premix[2]	0.15
Total	100.00
Calculated values	
Crude protein, %	12
Digestible energy, MJ/kg	13.455
Metabolizable energy, MJ/kg	12.985
Net energy, MJ/kg	10.125
Calcium, %	0.515
Available phosphorus, %	0.215
SID lysine, %	0.635
SID methionine, %	0.205
SID methionine+cystine, %	0.39
SID threonine, %	0.42
SID tryptophan, %	0.115
SID isoleucine, %	0.375
SID leucine, %	0.88
SID valine, %	0.435
Measured values	
Moisture, %	12.64
Crude protein, %	11.815
Ash, %	3.505
Crude fat, %	2.505
Calcium, %	0.54
Phosphorus, %	0.505
Gross energy, MJ/kg	16.04

1 Mineral premix provides per kg of basal diet: Fe, 150 mg; Cu, 20 mg; Zn, 150 mg; Mn, 20 mg; Se, 0.3 mg; I, 0.3 mg.

2 Vitamin premix provides kg of basal diet: vitamin A, 15,750 IU; vitamin D3, 3,500 IU; vitamin E, 26.2 mg; vitamin K, 3.5 mg; vitamin B1, 3.5 mg; vitamin B2, 8.8 mg, vitamin B12, 26.2 µg; vitamin B6, 17.5 mg; biotin, 0.14 mg; folic acid, 1.7 mg; nicotinamide, 35 mg.

Supplementary Table S2. Primer sequences used for RT-qPCR.

Gene name		Primer Sequence (5'-3')	GeneBank ID	Product size (bp)
MyHC I	Forward	CGTGGACTACAACATCATAGGC	NM_213855	146
	Reverse	CCTTCTCAACAGGTGTGTCG		
MyHC IIa	Forward	CATTGAGGCCCAGAATAGGC	AB025260	127
	Reverse	TGCTTCCGTCTTCACTGTCAC		
MyHC IIx	Forward	TTGACTGGGCTGCCATCAAT	AB025262	111
	Reverse	GCCTCAATGCGCTCCTTTTC		
MyHC IIb	Forward	GACTCTGGCTTTCCTCTTTGC	AB025261	101
	Reverse	GAGCTGACACGGTCTGGAAA		
GAPDH	Forward	ACACTGAGGACCAGGTTGTG	NM_001206359.1	98
	Reverse	GACGAAGTGGTCGTTGAGGG		

MyHC, myosin heavy chain; GAPDH, glyceraldehyde-3-phosphate dehydrogenase.

Supplementary Table S3. Transcriptomic summary.

Log2 fold change is DLY/TFB.

Result	Value
DEGs	989
TFB-high / DLY-high	673 / 316
DEG threshold	|log2FC| ≥ log2(1.5); BH-adjusted P ≤0.05
PCA PC1 / PC2	34.8% / 23.3%

Supplementary Table S4. Primary BH-significant metabolites.

1,412 features; 154 candidates (40 DLY-high, 114 TFB-high). Student BH <0.05: 16; Welch: five; Mann–Whitney: zero.

Putative annotation	Higher group	Student BH q	Welch BH q
1-Methylhistidine	TFB	0.04638	0.09779
Gamma-Glutamyl-Beta-Aminopropiononitrile	TFB	0.04638	0.07543
Pandamarilactam 3X	TFB	0.04638	0.104
(10Z,12E)-Hexadecadienoylcarnitine	TFB	0.04638	0.05905
4-Hexadecenoylcarnitine	TFB	0.04638	0.1173
(10Z,12Z)-Octadeca-10,12-Dienoylcarnitine	TFB	0.02583	0.05905
Ldgcc(11:0)	DLY	0.04638	0.104
(9Z,11E,13Z)-Octadeca-9,11,13-Trienoylcarnitine	TFB	0.03871	0.05905
Levulinic Acid	TFB	0.01054	0.01233
3-Hydroxy-Cis-5-Octenoylcarnitine	TFB	0.02583	0.04482
Tolmetin	TFB	0.04638	0.104
Levocarnitine	TFB	0.04638	0.07543
N-Acetyl-1-Methylhistidine	TFB	0.04638	0.07543
5-(4-Methyl-5-Oxo-2H-Furan-3-Yl)Pentanoic Acid	TFB	0.02583	0.03311
Safflor Yellow A	TFB	0.01985	0.03311
Chrysoeriol 7-O-(6"-Malonyl-Apiosyl-Glucoside)	TFB	0.01054	0.01629

Supplementary Table S5a. Selected GSEA results.

35 pathways meet BH-adjusted P <0.05. Positive NES: DLY; negative NES: TFB.

Pathway	NES	BH-adjusted P
Fructose and mannose metabolism	2.243	0.003866
Cholesterol metabolism	1.924	0.03438
Oxidative phosphorylation	-1.749	0.006654
Protein export	-1.743	0.03184
Glycolysis / Gluconeogenesis	1.689	0.05163
Motor proteins	1.579	0.03205
Central carbon metabolism in cancer	1.234	0.3988

Supplementary Table S5b. Histidine-pathway enrichment.

Background: 275 names; BH family: 122 pathways per method.

Candidate method	Mapped hits	BH-adjusted P
Student	9/14	0.002216
Welch	9/14	0.001392

Supplementary Table S6. Exploratory associations with $P < 0.05$.

Production-group-adjusted partial Spearman analysis: 12 animals and 180 tests. Seventeen unadjusted $P < 0.05$; two BH $q < 0.10$; none $q < 0.05$.

Feature	Trait	Partial ρ	P	BH q
N-Acetylhistamine	pH24h	0.8675	0.000541	0.0562
CYCS	a_45min	0.8630	0.000624	0.0562
NDUFB4	a_45min	0.7791	0.004710	0.2081
Homocarnosine	pH45min	-0.7720	0.005371	0.2081
PDHA1	a_45min	0.7632	0.006285	0.2081
Ribose 1,5-Bisphosphate	L_45min	0.7574	0.006937	0.2081
Ribose 1,5-Bisphosphate	a_45min	-0.7076	0.014859	0.3821
3-Phosphoglycerate	b_45min	-0.6846	0.020127	0.4285
2-Phospho-D-Glyceric Acid	Cooking_loss_pct	0.6713	0.023719	0.4285
CYCS	pH24h	0.6583	0.027664	0.4285
Homocarnosine	Shear_force_N	0.6455	0.031944	0.4285
PDHX	b_45min	0.6421	0.033165	0.4285
NDUFB4	pH45min	0.6317	0.037087	0.4285
COX7B	a_45min	0.6265	0.039149	0.4285
Ribose 1,5-Bisphosphate	pH45min	-0.6263	0.039227	0.4285
PPRC1	b_45min	0.6198	0.041958	0.4285
Histamine	Intramuscular_fat_pct	-0.6197	0.042015	0.4285

Supplementary Table S7. Mean differences and 95% confidence intervals.

Eight pigs/group, one/pen. Welch–Satterthwaite intervals verified against individual-data reanalysis by the authors. Percentage differences are percentage points.

Trait	Unit	TFB − DLY	95% CI	Welch P
Carcass weight	kg	-31.34	-34.78 to -27.90	<0.001
Dressing percentage	percentage points	-4.22	-5.69 to -2.75	<0.001
Carcass length	cm	-16.14	-19.75 to -12.53	<0.001
First rib backfat	cm	1.18	0.47 to 1.89	0.003
Last rib backfat	cm	0.65	-0.02 to 1.32	0.057
Last lumbar backfat	cm	1.37	0.88 to 1.86	<0.001
Average backfat	cm	1.06	0.54 to 1.58	0.000825
Loin eye area	cm^2	-17.54	-30.47 to -4.61	0.012
pH45min	unitless	-0.03	-0.15 to 0.09	0.594
pH24h	unitless	0.05	0.00037 to 0.09963	0.0486
L*45min	unitless	-1.00	-2.79 to 0.79	0.254
a*45min	unitless	0.79	-0.97 to 2.55	0.348
b*45min	unitless	0.68	-0.15 to 1.51	0.102
Drip loss	percentage points	-1.46	-2.38 to -0.54	0.004
Cooking loss	percentage points	-4.56	-8.41 to -0.71	0.025
Shear force	N	-11.96	-19.69 to -4.23	0.006
Intramuscular fat	percentage points	1.24	0.85 to 1.63	<0.001
